# Postprandial effects of a meal low in sulfur amino acids and high in polyunsaturated fatty acids compared to a meal high in sulfur amino acids and saturated fatty acids on stearoyl CoA-desaturase indices and plasma sulfur amino acids: a pilot study

**DOI:** 10.1186/s13104-020-05222-y

**Published:** 2020-08-10

**Authors:** Thomas Olsen, Cheryl Turner, Bente Øvrebø, Nasser E. Bastani, Helga Refsum, Kathrine J. Vinknes

**Affiliations:** 1 Department of Nutrition, Institute of Medical Biosciences, University of Oslo, 0372 Oslo, Norway; 2 Department of Pharmacology, University of Oxford, Oxford, OX1 3QT UK; 3 Øvrebø Nutrition, 0550 Oslo, Norway; 4 Institute of Medical Biosciences, Domus Medica, Sognsvannsveien 9, 0372 Oslo, Norway

**Keywords:** Sulfur amino acid restriction, Stearoyl CoA-desaturase, Methionine restriction, Cysteine, Vegan, Vegetarian

## Abstract

**Objective:**

The sulfur amino acid (SAA) cysteine is positively related, whereas polyunsaturated fatty acids (PUFAs) are inversely related to activity of the lipogenic enzyme stearoyl-CoA desaturase (SCD). High SCD activity promotes obesity in animals, and plasma activity indices positively associates with fat mass in humans. SCD may thus be a target for dietary intervention with SAA restriction and PUFA enrichment with unknown potential benefits for body composition. We randomized ten healthy individuals to a meal restricted in SAAs and enriched with PUFAs (Cys/Met_low_ + PUFA) (n = 5) or a meal enriched in SAA and saturated fatty acids (Cys/Met_high_ + SFA) (n = 5). We measured plasma SCD activity indices (SCD16 and SCD18) and SAAs response hourly from baseline and up to 4 h postprandial.

**Results:**

SCD16 was unchanged whereas SCD18 tended to increase in the Cys/Met_low_ + PUFA compared to the Cys/Met_high_ + SFA group (p_time*group interaction_ = 0.08). Plasma concentrations of total cysteine fractions including free and reduced cysteine decreased in the Cys/Met_low_ + PUFA compared to the Cys/Met_high_ + SFA group (both p_time*group interaction_ < 0.001). In conclusion, a meal low in SAA but high in PUFAs reduced plasma cysteine fractions but not SCD activity indices. This pilot study can be useful for the design and diet composition of future dietary interventions that targets SCD and SAA.

*Trial registration* ClinicalTrials.gov: NCT02647970, registration date: 6 January 2016

## Introduction

Methionine and cysteine are the main sulfur-containing amino acids (SAAs) involved in several crucial cellular mechanisms including methylation reactions and redox balance [1]. Specifically, methionine is the precursor of the universal methyl donor S-adenosylmethionine which participates in transmethylation reactions yielding a methylated product and S-adenosylhomocysteine. S-adenosylhomocysteine may subsequently be converted to homocysteine which in turn can undergo transsulfuration and form cysteine. Methionine and cysteine are mainly found in foods of animal origin whereas plant-based and vegetarian diets contain lower amounts and can lead to decreased SAA concentrations in circulation [2, 3]. A summary figure of SAA metabolism can be found in Additional file 1.

In humans, plasma concentrations of S-adenosylmethionine and total cysteine have been associated with fat mass [4–6], possibly by interaction with the lipo- and obesogenic enzyme stearoyl-CoA desaturase (SCD) [7, 8], which produces monounsaturated fatty acids for triglyceride synthesis and fat storage [9, 10]. Indeed, dietary restriction of methionine and cysteine reduced expression of SCD in the liver of mice [11]. Furthermore, SCD activity is also sensitive to the fatty acid composition of the diet [12], and particularly dietary intakes of polyunsaturated fatty acids (PUFAs). PUFA intakes repress transcription of SCD in the liver [13, 14] and high PUFA intakes has been associated with improved body composition [15]. Collectively, previous findings indicate that SCD is an appealing target for dietary interventions with SAA restriction and PUFA enrichment with the long-term aim to improve body composition and metabolic health. However, no human studies have been designed to maximally influence SCD with this particular dietary combination.

The primary aim of this postprandial pilot study was thus to evaluate whether test meals designed to maximally influence SCD affected activity indices of SCD in plasma. Because restriction of the SAAs in itself may have beneficial effects for metabolic health, a secondary aim was to assess the effects of the test meals on plasma concentrations of methionine and cysteine as well as related intermediates (Additional file 1: Figure S1). The results from this postprandial pilot study extend and complement a previously published 7-d pilot study [16], and will be used in the planning and design of a large-scale dietary intervention that aims to influence SCD activity indices with potential benefits for body composition and metabolic health.

## Main text

### Materials and methods

Detailed information on the screening and recruitment of participants has been published elsewhere [16]. Written informed consent was obtained from all participants. The study is reported according to the CONSORT guidelines. The study protocol was approved by the Regional Committee for Medical Research Ethics South East Norway (reference number: 2015/634) and was registered with the US National Library of Medicine Clinical Trials registry (ClinicalTrials.gov Identifier: NCT02647970).

Ten free-living, healthy individuals were recruited and randomized to receive a meal low in SAAs and high in PUFAs (Cys/Met_low_ + PUFA) (n = 5) or a control meal high in SAAs and saturated fatty acids (Cys/Met_high_ + SFA) (n = 5). The meal compositions were as follows: They were both vegan-based, excluding meat, fish, eggs, dairy products and certain plant-based foods but including butter in the Cys/Met_high_ + SFA group. All participants were given a powdered drink mix without SAAs (XMET XCYS Maxamaid ^®^ provided by Nutricia) but contained other essential and non-essential amino acids, carbohydrate, vitamins, minerals and trace elements. Powdered SAA (JoMar Labs, Scotts Valley, CA, USA) was added to the Cys/Met_high_ + SFA drink mix. Both meals included n-3 supplements [Triomar ^®^ containing 1.32 grams of n-3 and Møllers Dobbel Kapsel^®^ containing 0.4 g of n-3, vitamin A (139 µg), vitamin D (8.3 µg) and vitamin E (5.6 mg)]. Participants received the test meal at the study center. The meal consisted of a slice of focaccia topped with tomato and olive oil in the Cys/Met_low_ + PUFA group. The meal was identical in the Cys/Met_high_ + SFA group, but some of the plant oils in the focaccia were replaced with butter. The participants were considered blind to the interventions, but the addition of butter to the Cys/Met_high_ + SFA group may have complicated the blinding attempt. In addition to the meal, participants were given n-3 supplements and juice mixed with 25 g of the powdered drink mix. In total, 1.2 g of SAAs (0.8 g cysteine and 0.4 g methionine) were added to the powdered drink mix in the Cys/Met_high_ + SFA group. The energy content of the Cys/Met_low_ + PUFA meal was 687 kcals where 8.1% of energy (E%) came from SFAs and 11.3 E% from PUFAs (1.95 g n-3 and 5.85 g n-6), as well as 0.22 g of SAAs. The Cys/Met_high_ + SFA meal consisted of 725 kcals where 18.9 E% came from SFAs and 3.0 E% from PUFAs (0.75 g n-3 and 1.05 g n-6), and included 1.43 g of SAAs. Participants could drink up to 1 l of water up to 4 h after the test meal.

Venous blood samples were obtained at baseline and hourly up to 4 h postprandial by trained personnel at the Centre for Clinical Nutrition at the Department of Nutrition, University of Oslo. Main outcomes were plasma SCD activity indices and plasma concentrations of SAA and related metabolites including methionine, S-adenosylmethionine, S-adenosylhomocysteine, total homocysteine and its fractions, cystathionine, total cysteine and its fractions, total glutathione and its fractions and taurine. Plasma fatty acid profile and SAA and related metabolites were measured as previously described [16]. SCD indices were given as product/precursor-ratios (SCD-16; 16:1n-7/16:0, SCD-18; 18:1n-9/18:0). Participants were additionally asked to provide information on gastrointestinal discomfort, headaches and other potential side effects of the meals.

Baseline data were presented as medians (min, max) for continuous variables and counts (%) for categorical variables unless otherwise specified. The Wilcoxon rank-sum test was used to assess between-group differences for continuous variables at baseline. The responses to the test meal were compared using linear mixed model regression with the amino or fatty acid of interest as the outcome. The models included time, group and their interaction term (time × group) as fixed effects. The interaction term was included in order to test for differences between groups across time. Subjects were included as random effect to adjust for within-subject correlation. We derived estimated marginal means (EMMs) from the models and their 95% corresponding confidence intervals. The significance level was set to p < 0.05. Statistical analyses were carried out using R v.3.6.0 (the R Foundation for Statistical Computing, Vienna, Austria).

### Results

The study is reported according to the CONSORT guidelines and a flowchart of the participants is presented in Additional file 2. Participants were recruited and the intervention was implemented August–December 2016. Baseline characteristics for both groups are presented in Table 1. Both groups contained five participants and were generally similar in terms of weight, height, BMI and systolic blood pressure at baseline. Although not statistically significant, median age in the Cys/Met_low_ + PUFA group was higher, whereas diastolic blood pressure was lower compared with the Cys/Met_high_ + SFA group. All participants completed the postprandial assessment.

**Table 1:** Baseline characteristics of the participants

	Cys/Met_low_ + PUFA	Cys/Met_high_ + SFA	p-value
Participants, n	5	5	
Female	4	4	
Male	1	1	
Age, y	33 (22–38)	23 (20–34)	0.29
Weight, kg	65.0 (58.5–79.0)	64.6 (59.5–69.0)	0.69
Height, m	1.72 (1.68–1.77)	1.69 (1.59–1.75)	0.40
BMI, kg/m^2^	22.4 (20.7–26.1)	22.9 (20.8–23.9)	1.00
Systolic blood pressure, mm Hg	111 (99–124)	111 (104–137)	0.60
Diastolic blood pressure, mm Hg	58 (57–65)	67 (56–86)	0.21

Cys/Met_low_ + PUFA, meal with low contents of cysteine and methionine, and high contents of polyunsaturated fatty acids; Cys/Met_high_ + SFA, meal with high contents of cysteine, methionine and saturated fatty acids

Results for fatty acids 16:1n-7, 16:0, 18:1n-9 and 18:0, as well as SCD-16 and SCD-18 can be found in Table 2. No significant differences over time were observed between groups. However, we note a trend for a time × group interaction (p=0.08) for SCD-18 which tended to increase in the Cys/Met_low_ + PUFA group compared to the Cys/Met_high_ + SFA group.

**Table 2:** Estimated marginal means (95% confidence interval) for stearoyl-CoA desaturase activity indices, sulfur amino acids and related metabolites

	Group	Baseline	1 h	2 h	3 h	4 h	p for interaction
Fatty acids (μmol/L) and SCD							
16:0	Cys/Met_high_ + SFA	1690 (1310, 2070)	1750 (1370, 2120)	1820 (1440, 2190)	1990 (1610, 2360)	1980 (1600, 2350)	0.437
	Cys/Met_low_ + PUFA	1700 (1320, 2070)	1690 (1310, 2070)	1710 (1340, 2090)	1750 (1370, 2130)	1920 (1550, 2300)	
16:1n-7	Cys/Met_high_ + SFA	171 (105, 238)	184 (118, 251)	169 (103, 236)	187 (120, 253)	195 (129, 262)	0.429
	Cys/Met_low_ + PUFA	141 (74.3, 208)	138 (71, 204)	131 (64, 197)	133 (66.1, 199)	149 (82.1, 215)	
18:0	Cys/Met_high_ + SFA	609 (533, 686)	644 (568, 721)	663 (587, 740)	720 (644, 797)	723 (647, 799)	0.101
	Cys/Met_low_ + PUFA	621 (545, 697)	621 (545, 697)	621 (545, 697)	628 (552, 704)	693 (617, 769)	
18:1n-9	Cys/Met_high_ + SFA	1650 (1220, 2080)	1750 (1320, 2180)	1760 (1330, 2200)	1900 (1460, 2330)	1910 (1480, 2340)	0.439
	Cys/Met_low_ + PUFA	1770 (1340, 2200)	1760 (1330, 2190)	1800 (1370, 2230)	1860 (1420, 2290)	2120 (1690, 2550)	
SCD-16	Cys/Met_high_ + SFA	0.10 (0.07, 0.12)	0.10 (0.08, 0.13)	0.09 (0.07, 0.12)	0.09 (0.07, 0.12)	0.10 (0.07, 0.12)	0.788
	Cys/Met_low_ + PUFA	0.08 (0.06, 0.11)	0.08 (0.06, 0.11)	0.08 (0.05, 0.10)	0.0749 (0.05, 0.10)	0.08 (0.05, 0.10)	
SCD-18	Cys/Met_high_ + SFA	2.74 (2.09, 3.39)	2.73 (2.08, 3.38)	2.69 (2.04, 3.34)	2.63 (1.98, 3.28)	2.67 (2.02, 3.32)	0.077
	Cys/Met_low_ + PUFA	2.86 (2.21, 3.51)	2.85 (2.2, 3.5)	2.9 (2.25, 3.55)	2.95 (2.3, 3.6)	3.03 (2.38, 3.68)	
Sulfur amino acids, μmol/L							
Methionine	Cys/Met_high_ + SFA	25.1 (20.8, 29.3)	38.7 (34.5, 43.1)	34.1 (29.8, 38.3)	26.8 (22.6, 31.1)	23.4 (19.2, 27.7)	< 0.001
	Cys/Met_low_ + PUFA	23.5 (19.3, 27.8)	18.2 (13.9, 22.5)	15.1 (10.8, 19.4)	12.7 (8.46, 17.3)	13.2 (8.98, 17.5)	
SAM	Cys/Met_high_ + SFA	97.3 (86.6, 108)	109 (97.9, 119)	101 (90.5, 112)	95.7 (84.9, 106)	94.2 (83.4, 105)	< 0.001
	Cys/Met_low_ + PUFA	99.7 (88.9, 110)	88.6 (77.8, 99.3)	81.3 (70.5, 92.1)	78.1 (67.4, 88.9)	78.8 (68, 89.6)	
SAH	Cys/Met_high_ + SFA	14 (11.8, 16.2)	15.4 (13.2, 17.6)	14.4 (12.2, 16.6)	13.7 (11.5, 15.9)	13.2 (11.2, 15.4)	0.040
	Cys/Met_low_ + PUFA	13.8 (11.6, 15.9)	13.1 (10.9, 15.2)	12.1 (9.88, 14.2)	10.9 (8.68, 13)	10.6 (8.46, 12.8)	
SAM/SAH	Cys/Met_high_ + SFA	7.1 (6.2, 8.01)	7.19 (6.28, 8.09)	7.22 (6.32, 8.13)	7.14 (6.23, 8.04)	7.29 (6.39, 8.21)	0.424
	Cys/Met_low_ + PUFA	7.27 (6.37, 8.18)	6.8 (5.9, 7.71)	6.76 (5.86, 7.66)	7.21 (6.31, 8.12)	7.43 (6.53, 8.34)	
Total homocysteine	Cys/Met_high_ + SFA	8.61 (6.87, 10.4)	9.23 (7.49, 11.0)	8.27 (6.53, 10.2)	7.09 (5.35, 8.83)	6.71 (4.97, 8.45)	< 0.001
	Cys/Met_low_ + PUFA	7.98 (6.23, 9.72)	7.58 (5.84, 9.32)	7.74 (6.01, 9.48)	7.41 (5.67, 9.16)	7.39 (5.65, 9.13)	
Cystathionine, nmol/L	Cys/Met_high_ + SFA	156 (112, 199)	254 (211, 298)	281 (237, 324)	275 (231, 319)	246 (202, 289)	< 0.001
	Cys/Met_low_ + PUFA	127 (83.4, 171)	113 (69.3, 157)	98.3 (54.6, 142)	94.8 (51.2, 138)	98.3 (54.7, 142)	
Total cysteine	Cys/Met_high_ + SFA	228 (204, 252)	231 (207, 255)	231 (207, 256)	224 (199, 248)	225 (201, 250)	0.387
	Cys/Met_low_ + PUFA	235 (210, 259)	224 (199, 248)	218 (194, 243)	218 (194, 242)	216 (192, 241)	
Total GSH	Cys/Met_high_ + SFA	5.97 (4.29, 7.65)	5.7 (4.02, 7.39)	4.44 (2.75, 6.12)	5.72 (4.04, 7.41)	5.3 (3.62, 6.98)	0.169
	Cys/Met_low_ + PUFA	4.03 (2.35, 5.71)	5.37 (3.69, 7.05)	5.28 (3.59, 6.96)	5.35 (3.67, 7.03)	5.1 (3.42, 6.78)	
Taurine	Cys/Met_high_ + SFA	85.7 (65.3, 106)	86.8 (66.4, 107)	68.5 (48.1, 88.9)	77.1 (56.7, 97.5)	81 (60.6, 101)	0.082
	Cys/Met_low_ + PUFA	54.8 (34.3, 75.2)	58.5 (38.1, 78.9)	65.1 (44.7, 85.5)	69.6 (49.1, 90)	76.1 (55.7, 96.5)	

Cys/Met_high_ + SFA, meal with high contents of cysteine, methionine and saturated fatty acids; Cys/Met_low_ + PUFA, meal with low contents of cysteine and methionine, and high contents of polyunsaturated fatty acids; SAM, S-adenosylmethionine; SAH, S-adenosylhomocysteine; GSSG, oxidized glutathione; GSH, glutathione; SCD, steraoyl-CoA desaturase

The EMMs and corresponding confidence intervals and p-values for interaction between time × group for SAAs and related metabolites are presented in Tables 2 and 3. We observed linear decreases in plasma methionine and SAM in the Cys/Met_low_ + PUFA group after 4 h, whereas plasma concentrations increased for 2 h before returning to baseline concentrations in the Cys/Met_high_ + SFA group. Plasma concentrations of SAH decreased more in the Cys/Met_low_ + PUFA compared to the Cys/Met_high_ + SFA group. In contrast, plasma total homocysteine concentrations remained stable in the Cys/Met_low_ + PUFA group, whereas we observed a decrease in the Cys/Met_high_ + SFA group. Cystathionine decreased in the Cys/Met_low_ + PUFA group, whereas it increased sharply and remained elevated after 4 h in the Cys/Met_high_ + SFA group. No significant differences over time were observed for plasma total cysteine, total glutathione or taurine.

**Table 3:** Estimated marginal means (95% confidence interval) for fractions of total homocysteine, cysteine and glutathione

Metabolite, μmol/L	Group	Baseline	1 h	2 h	3 h	4 h	p for interaction
Homocystine, nmol/L	Cys/Met_high_ + SFA	21.6 (13.1, 30.2)	30.7 (22.1, 39.2)	24 (15.5, 32.5)	15.5 (6.99, 24)	13.5 (4.92, 22)	0.014
	Cys/Met_low_ + PUFA	15.8 (7.24, 24.3)	19.5 (11.0, 28.0)	17.3 (8.81, 25.9)	14.4 (5.85, 22.9)	12.4 (3.89, 20.9)	
Reduced homocysteine, nmol/L	Cys/Met_high_ + SFA	130 (92.4, 167)	188 (151, 226)	185 (148, 223)	154 (117, 191)	150 (113, 188)	0.014
	Cys/Met_low_ + PUFA	150 (113, 187)	194 (157, 231)	117 (80, 155)	101 (63.8, 138)	97.4 (60.1, 135)	
Protein-bound homocysteine	Cys/Met_high_ + SFA	6.36 (5.01, 7.7)	6.51 (5.16, 7.85)	5.60 (4.25, 6.94)	4.63 (3.29, 5.98)	4.47 (3.12, 5.81)	0.002
	Cys/Met_low_ + PUFA	5.74 (4.4, 7.09)	5.54 (4.2, 6.89)	5.79 (4.44, 7.13)	5.61 (4.26, 6.95)	5.59 (4.25, 6.94)	
Unbound homocysteine	Cys/Met_high_ + SFA	2.25 (1.78, 2.73)	2.73 (2.25, 3.2)	2.67 (2.2, 3.14)	2.46 (1.99, 2.93)	2.24 (1.77, 2.72)	0.001
	Cys/Met_low_ + PUFA	2.23 (1.76, 2.7)	2.04 (1.56, 2.51)	1.95 (1.48, 2.43)	1.80 (1.33, 2.28)	1.80 (1.32, 2.27)	
Cystine	Cys/Met_high_ + SFA	43.1 (37.4, 48.8)	46.9 (41.2, 52.6)	50 (44.3, 55.7)	47.6 (41.9, 53.3)	47.1 (41.4, 52.9)	0.001
	Cys/Met_low_ + PUFA	39.3 (33.6, 45)	41.8 (36, 47.5)	38.4 (32.7, 44.1)	37.3 (31.5, 43)	35.9 (30.2, 41.6)	
Reduced cysteine	Cys/Met_high_ + SFA	9.85 (7.18, 12.5)	12.5 (9.87, 15.2)	16.6 (14, 19.3)	18.5 (15.8, 21.1)	17.4 (14.8, 20.1)	< 0.001
	Cys/Met_low_ + PUFA	10.5 (7.87, 13.2)	11.6 (8.92, 14.2)	8.22 (5.56, 10.9)	7.47 (4.8, 10.1)	7.73 (5.07, 10.4)	
Protein-bound cysteine	Cys/Met_high_ + SFA	107 (88.7, 125)	101 (82.9, 119)	94.8 (76.6, 113)	83.9 (65.7, 102)	87 (68.8, 105)	0.270
	Cys/Met_low_ + PUFA	115 (97.2, 134)	113 (94.8, 131)	113 (95.3, 132)	112 (94.2, 131)	110 (92, 128)	
Unbound cysteine	Cys/Met_high_ + SFA	121 (108, 134)	130 (117, 143)	136 (123, 150)	140 (127, 153)	138 (125, 151)	< 0.001
	Cys/Met_low_ + PUFA	119 (106, 132)	111 (97.5, 124)	105 (91.5, 118)	105 (92.1, 119)	106 (93, 119)	
GSSG, nmol/L	Cys/Met_high_ + SFA	39.9 (28.9, 50.9)	26.9 (15.9, 37.9)	27.4 (16.4, 38.5)	33.1 (22.1, 44.2)	28.6 (17.5, 39.6)	0.098
	Cys/Met_low_ + PUFA	35.7 (24.7, 46.7)	30.4 (19.4, 41.4)	40.8 (29.8, 51.8)	33.1 (22.1, 44.1)	31.2 (20.2, 42.2)	
Reduced GSH	Cys/Met_high_ + SFA	2.76 (2.18, 3.34)	2.71 (2.13, 3.29)	2.47 (1.89, 3.05)	2.48 (1.9, 3.06)	2.36 (1.78, 2.94)	0.814
	Cys/Met_low_ + PUFA	2.58 (2, 3.16)	2.44 (1.86, 3.02)	2.56 (1.98, 3.14)	2.58 (2, 3.16)	2.63 (2.05, 3.21)	
Protein-bound GSH	Cys/Met_high_ + SFA	1.41 (0.14, 2.67)	2.04 (0.78, 3.30)	0.97 (− 0.29, 2.23)	2.04 (0.78, 3.31)	1.48 (0.22, 2.74)	0.875
	Cys/Met_low_ + PUFA	0.337 (− 0.93, 1.6)	1.21 (− 0.05, 2.48)	1.01 (− 0.25, 2.27)	1.44 (0.18, 2.71)	1.01 (− 0.25, 2.28)	
Unbound GSH	Cys/Met_high_ + SFA	4.56 (3.71, 5.41)	3.66 (2.81, 4.51)	3.47 (2.62, 4.32)	3.68 (2.83, 4.53)	3.82 (2.97, 4.67)	0.030
	Cys/Met_low_ + PUFA	3.69 (2.84, 4.54)	4.16 (3.31, 5.01)	4.26 (3.42, 5.11)	3.91 (3.06, 4.76)	4.08 (3.23, 4.93)	

Cys/Met_high_ + SFA, meal with high contents of cysteine, methionine and saturated fatty acids; Cys/Met_low_ + PUFA, meal with low contents of cysteine and methionine, and high contents of polyunsaturated fatty acids; GSSG, oxidized glutathione; GSH, glutathione

We observed significant differences between groups over time for all fractions of total homocysteine in plasma. In particular, homocystine and reduced homocysteine were decreased in plasma of both groups after 4 h. The decrease was more pronounced for Cys/Met_high_ + SFA group for homocystine, whereas concentrations of reduced homocysteine decreased more in the Cys/Met_low_ + PUFA group. For fractions of tCys, unbound cysteine including reduced cysteine and cystine decreased in the Cys/Met_low_ + PUFA compared to the Cys/Met_high_ + SFA group. Except for unbound glutathione which increased in the Cys/Met_low_ + PUFA compared to the Cys/Met_high_ + SFA group, no significant differences were observed for fractions of total glutathione.

No gastrointestinal discomforts or other harmful effects were reported by the participants.

### Discussion

Although we did not observe meaningful effects of the intervention meals on plasma SCD activity indices, this pilot study is the first to show that fractions of plasma total cysteine, including unbound cysteine (cystine and reduced cysteine), decrease after a meal low in the SAAs methionine and cysteine, and high in PUFAs compared to a meal high in methionine, cysteine and SFAs. This may seem intuitive, however, we have previously shown that there were no changes in plasma concentrations of total cysteine, whereas we observed a surprising, but clear, trend of increased cystine after a 7-d intervention consisting of a diet with a similar composition as the meals in the present study [16]. These contrasting observations indicate differential regulation of plasma total cysteine depending on the duration of the dietary restriction.

Similar to cysteine, we observed no changes in S-adenosylmethionine after a 7 d diet with a similar composition in a previous investigation [16]. Thus, it appears that fractions of plasma total cysteine and S-adenosylmethionine are differentially regulated depending on the duration of restriction. We will seek to elucidate the relevance of these mechanisms in future, full-scale human interventions, as they may be of importance considering the apparent association of total cysteine and S-adenosylmethionine with obesity [5, 17, 18]. Notably, it raises the possibility of whether intermittent instead of persistent restriction of methionine and cysteine may be an alternative in the longer term to achieve the health benefits demonstrated by SAA restriction in animal models [19, 20].

The changes in methionine and cystathionine were in line with a previous 7-d intervention [16], whereas no meaningful effects were observed for SCD activity indices, indicating that the PUFA contents may not have been high enough to achieve an effect. Few randomized controlled dietary interventions have targeted plasma SCD activity indices, but one 15 d study reported that SCD activity indices are affected by PUFA-rich foods compared to carbohydrate-rich control foods in a similar population to ours [21], indicating that a single meal might not be sufficient to detect relevant effects. Also, in the present study we cannot exclude the possibility that PUFA intakes among the participants were already high and that the run-in period of 7 d prior to the meal test was not sufficient for wash-out.

In addition to the issues related to duration of the intervention and the population under study, other aspects will inform the planning and design of a full-scale trial. First, considering that SCD is thought to be involved in obesity development, a study sample more representative for the target population should be recruited. In a study sample of participants with overweight and obesity, it would be reasonable to expect lower diet quality and elevated plasma SCD activity indices and SAAs at baseline [7, 19, 22, 23], and we would thus expect a clearer effect of the test meals used in the present pilot study. A second point to consider is the sample size and outcome measures of a future large-scale. Based on the literature, it is difficult to determine a clinically meaningful reduction in SCD, making sample size calculations a challenge. One previous 15 d study showed significant effects of daily PUFA-rich salmon intakes on plasma SCD activity indices with a relatively small n (n = 30) [21]. However, the primary outcome in a full-scale study is likely to be some measure of body composition or clinically meaningful metabolic parameter, which is easier to use for power calculations. Until such a trial can be justified however, it must be determined how much we can expect SCD and plasma SAAs to change in response to dietary intervention and whether this response can mediate beneficial effects on body composition and other parameters of metabolic health. Although some short, preliminary pilot studies have been performed [16, 24], a continued research effort is required to further justify the relevance of a large-scale trial.

## Limitations

The results from this study is limited by the small sample size, which reduce the statistical power to detect effects. We point out that this was a pilot study where the results are intended to be used in the planning of full-scale studies. In addition, we acknowledge that a 2 × 2 factorial design might have been a more suitable alternative to distinguish the effects of SAA and PUFA interventions. However, we stress that the aim of this study was in part to create optimal meals to affect both plasma SCD activity indices and SAA in line with the existing literature. This particular aspect will be thoroughly evaluated in the design of the full-scale study. Finally, because we only have data up until 4 h, the full effect of the meals on the measured plasma and serum parameters may not have been captured.

In conclusion, we have shown that a single meal low in SAA but high in PUFAs reduced plasma cysteine fractions but not SCD activity indices. Further studies should aim to elucidate the mechanisms responsible for short- and long-term control of plasma cysteine concentrations.

## Supplementary information

**Additional file 1.** Contains a schematic overview of sulfur amino acid metabolism.

**Additional file 2.** Contains the CONSORT figure.

## Data Availability

The datasets used and analyzed during the current study are available from the corresponding author on reasonable request.

## Abbreviations

Cys/Met_low_ + PUFA: Meal low in methionine and cysteine and high in polyunsaturated fatty acids
Cys/Met_high_ + SFA: Meal high in methionine, cysteine and saturated fatty acids
PUFA: Polyunsaturated fatty acids
SFA: Saturated fatty acids
SAA: Sulfur amino acids
SCD: Stearoyl CoA-desaturase
