# Supplementary material for: Postprandial effects of a meal low in sulfur amino acids and high in polyunsaturated fatty acids compared to a meal high in sulfur amino acids and saturated fatty acids on stearoyl CoA-desaturase indices and plasma sulfur amino acids: a pilot study
Source: BMC Res Notes. 2020 Aug 10;13:379. doi: 10.1186/s13104-020-05222-y (PMC7419218; doi:10.1186/s13104-020-05222-y)
Supplement: Supplementary file 1 — Additional file 1. Contains a schematic overview of sulfur amino acid metabolism. [file 13104_2020_5222_MOESM1_ESM.docx]

**Additional File 1 Figure S1:** The metabolism of sulfur amino acids. Double arrow heads indicate omitted steps for simplicity. Abbreviations; MAT, Methionine adenosyltransferase; MTs, methyltransferases; MS, methionine synthase; BHMT, betaine-homocysteine methyltransferase; SAHH, S-adenosylhomocysteine hydrolase; CBS, cystathionine beta-synthase; CGL, cystathionine gamma-lyase; CDO, cysteine dioxygenase; GGCS, gamma-glutamylcysteine synthase.
