# Supplementary figures and images for: Postprandial effects of a meal low in sulfur amino acids and high in polyunsaturated fatty acids compared to a meal high in sulfur amino acids and saturated fatty acids on stearoyl CoA-desaturase indices and plasma sulfur amino acids: a pilot study
Source: BMC Res Notes. 2020 Aug 10;13:379. doi: 10.1186/s13104-020-05222-y (PMC7419218; doi:10.1186/s13104-020-05222-y)

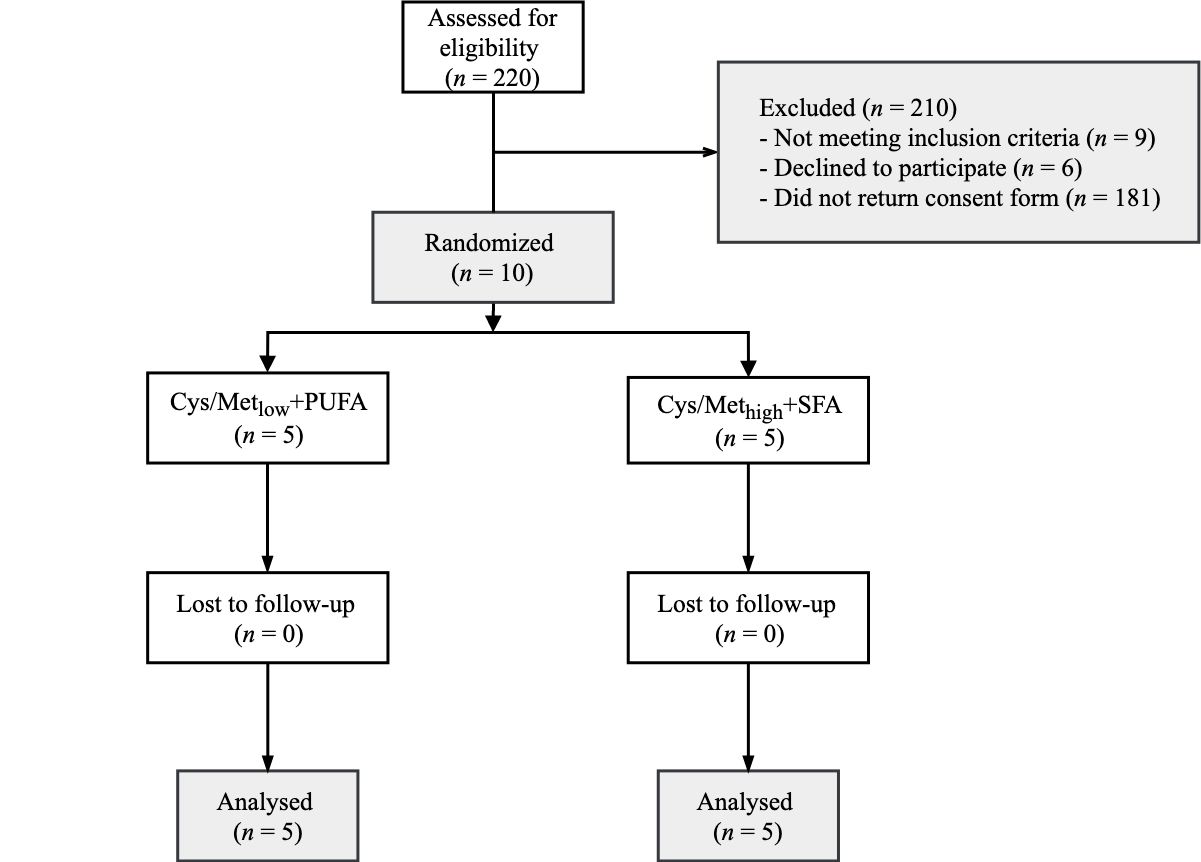


**Additional File 2 Figure S2:** CONSORT flow diagram of participants.

Supplement: Supplementary file 2 — Additional file 2. Contains the CONSORT figure. [file 13104_2020_5222_MOESM2_ESM.docx]
